# Effect of Virtual Reality on Postural and Balance Control in Patients with Stroke: A Systematic Literature Review

**DOI:** 10.1155/2016/7309272

**Published:** 2016-12-07

**Authors:** Ling Chen, Wai Leung Ambrose Lo, Yu Rong Mao, Ming Hui Ding, Qiang Lin, Hai Li, Jiang Li Zhao, Zhi Qin Xu, Rui Hao Bian, Dong Feng Huang

**Affiliations:** Department of Rehabilitation Medicine, Guangdong Engineering and Technology Research Center for Rehabilitation Medicine and Translation, The First Affiliated Hospital, Sun Yat-sen University, Guangzhou 510080, China

## Abstract

*Objective*. To critically evaluate the studies that were conducted over the past 10 years and to assess the impact of virtual reality on static and dynamic balance control in the stroke population.* Method*. A systematic review of randomized controlled trials published between January 2006 and December 2015 was conducted. Databases searched were PubMed, Scopus, and Web of Science. Studies must have involved adult patients with stroke during acute, subacute, or chronic phase. All included studies must have assessed the impact of virtual reality programme on either static or dynamic balance ability and compared it with a control group. The Physiotherapy Evidence Database (PEDro) scale was used to assess the methodological quality of the included studies.* Results*. Nine studies were included in this systematic review. The PEDro scores ranged from 4 to 9 points. All studies, except one, showed significant improvement in static or dynamic balance outcomes group.* Conclusions*. This review provided moderate evidence to support the fact that virtual reality training is an effective adjunct to standard rehabilitation programme to improve balance for patients with chronic stroke. The effect of VR training in balance recovery is less clear in patients with acute or subacute stroke. Further research is required to investigate the optimum training intensity and frequency to achieve the desired outcome.

## 1. Introduction

Stroke is the leading cause of death and disability worldwide [1]. The worldwide prevalence was reported to be 33 million in 2010 [2]. The mortality rate of stroke had fallen by 35.8% over the past decade and, yet, approximately 795000 people in the United States continue to experience an episode of stroke [3]. China has the highest prevalence in the world [4, 5]. An epidemiological study suggested that the incidence of stroke in China was 116 to 229/100,000 person-years, leaving about 75% of individuals with motor dysfunction and 40% with severe disability [6]. Stroke survivors often have deficit in motor control which contributed to reduced balance [7, 8], postural control and mobility [9], and reduced proprioception [10]. It is known that stroke status can affect activities of daily living and social participation [11]. The ability to maintain upright posture is essential to maintain balance and balance is negatively affected by postural control [12]. Although the majority of stroke patients recover gait function after rehabilitation, balance and gait deficit persist through the chronic stage [13]. Reduced static balance and dynamic balance are major risk factors of falls [14, 15] and limit the ability to perform activities of daily living [16].

Despite the importance of balance, studies that focused on postural control are lacking in comparison with limbs or gaits balance rehabilitation. Several interventions regarding balance and trunk control had been investigated, including weight-shift training on an unstable surface [17], balance control training [18], and gait training with rhythmic auditory stimulation [19]. The majority of published studies reported positive results but traditional rehabilitation programmes tend to be tedious and resource-intensive and require specialized facilities or equipment [20–22]. Given that stroke prevalence is rising every year, there is an urgent need to identify intervention methods which are both cost-effective and safe.

Virtual reality (VR) has a prominent role in promoting functional recovery after stroke. It has the potential to deliver the effective intervention at low cost [32]. VR provides enriched motivational training and goal-orientated tasks which improve patients' adherence to programme [33, 34]. It can integrate multisensory stimulation of visual, auditory, tactile, and somatosensory systems to provide a realistic environment [35]. Previous studies indicated that it might be more effective in improving dynamic balance control and preventing falls in subacute and chronic stroke patients compared to conventional therapy [36]. However, it may not always be superior to conventional therapy [37]. This is likely due to the heterogeneity of VR programme and the sample characteristics and outcome measures being used. Given that there has been rapid development of VR programme over the past decades, it is essential to review the evidence of VR on balance control in stroke survivors to enable clinicians to have an up-to-date understanding of the clinical applications in posture and balance ability. The aim of the systematic review is to critically evaluate the evidence of VR on improving static balance and dynamic balance ability in the stroke population.

## 2. Materials and Methods

### 2.1. Search Strategy

An extensive retrieval of the scientific articles published between January 2006 and December 2015 was conducted. Databases searched were PubMed, Scopus, and Web of Science. References from retrieved articles were manually checked for further suitable studies. The literature search used keywords terms and the principle of combining free word search based on the search engine, subject headings, and keywords. Databases were searched using the following key terms: (virtual reality OR virtual environment) AND (stroke OR hemiplegia OR cerebrovascular accident) AND balance. Titles and abstracts were displayed and screened by the two authors (LC and WL) to identify relevant studies. The authors then reviewed the titles and abstracts to assess whether the studies met the predetermined inclusion criteria.

### 2.2. Study Selection

Studies that met the following inclusion criteria were eligible:

- Published in English language
- Randomized controlled trials (RCTs) that compared preintervention and postintervention values
- Involved adult patients with hemiparesis following stroke during acute, subacute, and chronic phase
- Investigated any form of immersive or nonimmersive VR training therapy that aimed to improve balance control after stroke
- Utilized specific outcome measures to assess balance

The two authors assessed the studies for inclusion criteria independently. Any disagreement in study selection was resolved in consensus meetings.

### 2.3. Study Quality Assessment

The Physiotherapy Evidence Database (PEDro) scale was used to evaluate the methodological quality of the studies that met the inclusion criteria [38]. The PEDro is a reliable quality assessment scale [39] with 11-item scale developed to assess the methodological quality and internal validity of the RCTs. The items are as follows: (1) eligibility criteria were specified; (2) subjects were randomly allocated to groups; (3) allocation was concealed; (4) the groups were similar at baseline regarding the most important prognostic indicators; (5) there was blinding of all subjects; (6) there was blinding of all therapists who administered the therapy; (7) there was blinding of all assessors who measured at least one key outcome; (8) measures of at least one key outcome were obtained from more than 85% of the subjects initially allocated to groups; (9) all subjects for whom outcome measures were available received the treatment or control condition as allocated or, where this was not the case, data for at least one key outcome was analyzed by “intention to treat”; (10) the results of between-group statistical comparisons are reported for at least one key outcome; (11) the study provides both point measures and measures of variability for at least one key outcome. Except for item (1) which refers to external validity, the rest of the items receive either a “yes” or a “no” score. A study can receive the maximum score of 10 [39]. Foley's quality assessment was used to interpret the score [40]. Studies were rated as excellent to poor based on the following classification: 9-10 is considered to be excellent; 6–8 is considered to be good; 4-5 is considered to be fair; and a score of less than 4 is considered to be poor. The quality assessment (PEDro scale) was conducted independently by two of the authors (LC and WL). The scores from each assessor were cross-checked. Any disagreement was resolved in consensus meetings with the third assessor (YYM).

### 2.4. Data Collection

Data extracted were age, time since stroke onset, intervention and control protocol, frequency and duration of interventions, outcome measures, main results, and the PEDro score.

## 3. Results

### 3.1. Data Synthesis

**Figure 1:**
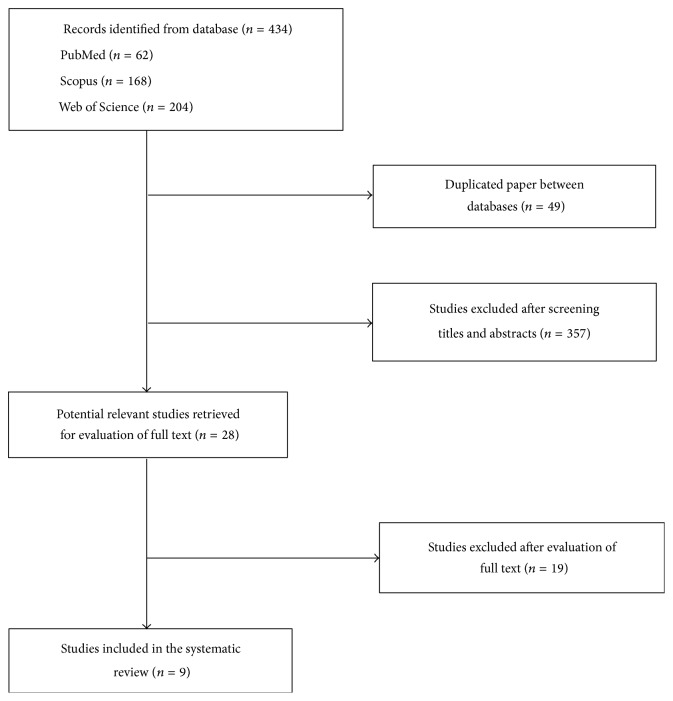
Flow diagram of study selection.

The initial search retrieved a total of 434 articles from the databases. After removing duplicates, 385 potential articles were identified. The authors independently evaluated the titles and abstracts. Finally, nine articles met all the inclusion criteria and were included in this review [23–31].  Figure 1 presents the flow diagram for the literature search process.

### 3.2. Study Characteristics

A summary of the included studies is presented in Table 1.

*(a) Population. *Seven studies had sample size of less than 30 participants [23–26, 31] and two studies had over 50 participants [29, 30]. All studies included male and female participants. The mean age of participants ranged between 52 and 66 years among the included studies. The mean timing of intervention was between 35 days and 3 years after stroke.

*(b) Intervention*. Two studies utilized the Interactive Rehabilitation Exercise software (IREX) VR games [23, 29]. Three studies utilized VR treadmill training [24, 26, 28]. The other three studies utilized commercially gaming systems of Nintendo Wii Sport [27], Wii Fit [25, 27, 30], and PC games EyeToy: Play 2 and Xbox Kinetic [27]. The duration of VR programme varied between 20 minutes and one hour per session. The total number of sessions varied between 9 and 20. 

*(c) Control*. Five studies provided the same dosage of treatment in both intervention group and control group [24, 25, 28–30]. One study included a control group without intervention [27]. Three studies provided VR balance training in addition to conventional therapy [23, 25, 31]. Three studies provided VR balance training with treadmill balance training without VR [24, 26, 28]. 

*(d) Outcome Measures. *All studies recorded more than one outcome measure at baseline and after intervention. A range of outcome measures was used to measure static balance, dynamic balance, walking balance, gait, and mobility. Seven studies used Berg Balance Scale (BBS). Six studies used Timed Up and Go test (TUG). Five of the studies used force platform to evaluate dynamic balance and static balance. All studies, except one [27], showed significant improvement in balance and gait outcomes. VR training group demonstrated greater improvement in gait speed and gait parameters when compared to control groups.

### 3.3. Quality Assessment

All included studies have quality score ranging from 4 to 9 points. Of the included studies, two RCTs were graded as fair. Six RCTs [25–29, 31] were graded as good and one RCT was graded as excellent [23].  Table 2 illustrates the PEDro assessment of all included studies.

## 4. Discussion

Reduced balance and postural control is a major contributor to functional limitations and barriers to perform activities of daily living in patients with stroke [41]. This study reviewed existing evidence on VR training on balance and postural control.

Fair-to-excellent quality evidence supports the positive impact of VR training in improving static balance and dynamic balance in patients with stroke. Seven out of the nine included studies concern chronic stroke patients. The strongest evidence, resulting from the excellent scoring based on PEDro scale [23], supports the fact that VR balance training is an effective adjunct to routine physiotherapy to improve dynamic balance and static balance in patients with chronic stroke. Good evidence [26, 28] indicated VR augment tradition physiotherapy or treadmill exercises programme to improve dynamic balance. However, VR training on its own does not appear to have any benefit in patients with chronic stroke as indicated in the moderate quality study by Fritz et al. [27]. They indicated no statistically significant difference in any balance-related outcome measure between or within groups. This finding casts some doubt as to whether VR training would be an effective method to deliver balance training in a home setting without therapists' input. Further research is recommended to confirm or refute this concern.

Moderate evidence [25, 28] indicated that VR training did not have significant impact on static balance during standing as measured by postural sway pressure displacement. However, lack of difference did not appear to be related to or have impact on dynamic balance or gait recovery. Only one high-quality study [23] reported improvement in static standing balance after VR training. Although postural sway is commonly believed to be the basis of feedback system to recalibrate the postural control system relating to the center of gravity, it is debatable whether static balance is related to dynamic balance or functional abilities [42]. Previous studies on body weight supported treadmill training also reported improvement in balance after interventions [43] but did not report significant effect on standing postural sway [44]. The finding from this review challenges the common beliefs that a decrease in postural sway is negatively related to functional improvement.

Two studies of moderate and fair quality concerned the balance recovery during acute and subacute stages [29, 30]. Both studies reported significant improvements in dynamic balance within group and between groups. However, the two studies used different outcome measures to assess balance ability; thus firm conclusion could not be drawn with regard to the effectiveness of VR on balance recovery during the acute and subacute stages. One study [29] included VR training in both intervention and control groups with the same intervention carried out in different positions. Thus, it is difficult to separate the coupling effect of VR and balance improvement. The other study [30] suffered from methodological bias of lack of blinding, concealed allocation, and missing data. The impact of VR on balance recovery during the early phase of stroke remains unclear.

All of the included studies have similar methodological weakness. None of the included studies performed sample size calculation for the primary outcome measure and the sample size could be considered to be small. Therefore, the result of the included studies is likely to contain type one error. The relatively small sample size also lacks external validity and therefore the reported findings may not necessary be applicable to the wider population. The intensity and duration of intervention also varied widely between studies and no justification was provided in any of the studies. The number of sessions varied between 9 and 20 and the duration differed in a range from 20 minutes to 1 hour. Several studies included VR training in addition to routine rehabilitation without increasing the equivalent amount of input in the control group. This reduced the rigorousness of the study as no firm conclusion could be drawn on whether the observed benefits were related to the additional training or due to the VR intervention. Further research is essential to identify the optimum “dosage” of recovery programme. Seven studies had risk of bias due to the lack of blinding of either therapist or participants [24–28, 30, 31].

All the included studies utilized different VR system, ranging from sophisticated laboratory-based device to standard commercial gaming device. While they all come under the category of “VR,” it is likely that there are differences between the VR devices and therefore there may exist different level of effectiveness in balance ability. As indicated in another review [45], it is difficult to determine the impact of each individual system on the outcomes. It is therefore not possible to draw conclusion as to which type of VR system is the most cost-effective.

## 5. Conclusion

Overall, there is some moderate evidence suggesting that VR training is an effective adjunct to standard rehabilitation programme for patients with chronic stroke. The effect of VR training on balance recovery is less clear in patients with acute or subacute stroke. Further research is required to investigate the optimum training intensity and frequency to achieve the desired outcome. It would also be beneficial for clinician to understand which training device may be more beneficial for patients in each subgroup.

## Figures and Tables

**Table 1:** Characteristics of included primary studies.

Citation year	Participants	Intervention	Frequency of stimulation	Outcome measure	Main results
Kim et al., 2009 [23]	*N* = 24 Mean age: 52.0 years; time since stroke: 2.1 years	E: IREX VR games + PT C: PT	16 sessions, 30 minutes/day, 4 days/week, 4 weeks.	BBS, BPM, 10 m walking test, MMAS, and sway angle during static standing and walking	BBS, MMAS, and postural sway angles during static standing and walking were higher in intervention group than in control group (*P* < 0.05). Cadence, step time, step length, and stride length were higher in intervention group than in control group (*P* < 0.05).

Yang et al., 2011 [24]	*N* = 14 Mean age: 61.0 years; time since stroke: 16.7 months	E: VR treadmill + PT OT C: traditional treadmill training + PT OT	9 sessions, 20 minutes/day, 3 days/week, 3 weeks.	Postural sway excursion during standing, sit-to-stand, and level walking	Sway excursion in medial-lateral direction and sit-to-stand transfers in intervention group were significantly lower compared to control group (*P* < 0.05).

Cho et al., 2012 [25]	*N* = 24 Mean age: 64.2 years; time since stroke: >6 months	E: Nintendo Wii Fit + PT OT C: PT OT	18 sessions, 30 minutes/day, 3 days/week, 6 weeks.	BBS, TUG, and postural sway velocity during standing	BBS and TUG in intervention group were significantly higher in the intervention group than in control group (*P* < 0.05). Postural sway velocities were not statistically different between the two groups (*P* > 0.05).

Cho and Lee, 2013 [26]	*N* = 14 Mean age: 64.9 years; time since stroke: 300.4 days	E: VR-based treadmill using real-world video recording C: non-VR treadmill	18 sessions, 30 minutes/day, 3 days/week, 6 weeks.	BBS, TUG, and gait performance	Greater improvement in the BBS, TUG, velocity, and cadence in intervention group compared to control group (*P* < 0.05).

Fritz et al., 2013 [27]	*N* = 30 Mean age: 66.1 years; time since stroke: 3.1 years	E: Nintendo Wii Sports and Wii Fit and PS games EyeToy: Play 2 and Kinetic C: no intervention	20 sessions, 50–60 minutes/day, 4 days/week, 5 weeks.	BBS, FMA, gait performance, 6-minute walk test, and 3-meter walk	No statistically significant differences between or within groups (*P* > 0.05).

Cho and Lee, 2014 [28]	*N* = 30 Mean age: 64.7 years; time since stroke: 437.0 days	E: VR-based treadmill using real-world video recording + PT, OT, and FES C: non-VR treadmill + PT, OT, and FES	18 sessions, 30 minutes/day, 3 days/week, 6 weeks.	BBS, TUG, postural sway, and gait temporospatial parameters	Significant improvements were reported in BBS, postural sway during gait, and gait parameters in both groups (*P* < 0.05). Greater improvements were reported in intervention group compared to control group. No difference was reported in postural sway during static standing (*P* > 0.05).

McEwen et al., 2014 [29]	*N* = 59 Mean age: 64.1 years; time since stroke: 34.8 days	E: IREX VR games in standing C: IREX VR games in sitting	10 to 12 sessions, 20 minutes/day, 3 weeks.	TUG, TMWT, and Chedoke-McMaster Stroke Assessment scale leg domain	Both groups had improvement in all outcome measures. Chedoke-McMaster leg domain score was significantly higher in intervention group than in control group (*P* < 0.05).

Morone et al., 2014 [30]	*N* = 50 Mean age: 60.2 years; time since stroke: 51.5 days	E: Wii Fit + PT C: usual balance therapy + PT	12 sessions, 20 minutes/day, 3 days/week, 4 weeks.	BBS, BI, and 10 m walking test recorded at baseline, after intervention, and at one-month follow-up	BBS, BI, and 10 m walking test were significantly higher in intervention group than in control group (*P* < 0.05). The difference was maintained at one-month follow-up.

Lloréns et al., 2015 [31]	*N* = 20 Mean age: 56.7 years; time since stroke: 497.6 days	E: VR step training with PT C: PT	20 sessions, 1 hour/day, 5 days/week, 4 weeks.	BBS, the balance and gait subscales of the Tinetti Performance-Oriented Mobility Assessment, BBAC, and the 10 m walking test	Both groups have significant improvement in BBS and 10 m walking test (*P* < 0.01). Greater improvements were reported in intervention group compared to control group.

BBA: Brunel Balance Assessment Category; BBS: Berg Balance Scale; BI: Barthel Index; BPM: Balance Performance Monitor; C: control group; COP: center of pressure; E: experimental group; FAC: Functional Ambulation Categories; FES: functional electrical stimulation; FMA: Fugl-Meyer Assessment; IREX: Interactive Rehabilitation Exercise software; MMAS: Modified Motor Assessment Scale; OT: occupational therapy; PT: physical therapy; RCT: randomized controlled trial; TMWT: Two-Minute Walk Test; TUG: Timed Up and Go test; VR: virtual reality.

**Table 2:** Physiotherapy Evidence Database scale criteria and scores for the trails.

	Kim et al., 2009 [23]	Yang et al., 2011 [24]	Cho et al., 2012 [25]	Cho and Lee, 2013 [26]	Fritz et al., 2013 [27]	Cho and Lee, 2014 [28]	McEwen et al., 2014 [29]	Morone et al., 2014 [30]	Lloréns et al., 2015 [31]
Random allocation	1	1	1	1	1	1	1	1	1
Concealed allocation	0	0	0	1	1	1	0	0	1
Baseline comparability	1	0	1	1	0	1	1	0	1
Subject blinded	1	0	0	0	0	0	1	0	0
Therapists blinded	1	0	0	0	0	0	0	0	0
Assessor blinded	1	1	0	1	1	1	1	1	1
Data for at least 1 outcome from >85% of subjects	1	0	1	0	1	0	0	0	1
No missing data or, if missing, intention-to-treat analysis	1	0	1	1	1	1	0	1	1
Between-group analysis	1	1	1	1	1	1	1	1	1
Point estimates and variability	1	1	1	1	0	1	1	1	1

Total score (/10)	9	4	6	7	6	7	6	5	8

*1 = yes; 0 = no*.
